# Evaluation of the national sobriety checkpoints program in Mexico: a difference-in-difference approach with variation in timing of program adoption

**DOI:** 10.1186/s40621-022-00407-4

**Published:** 2022-11-21

**Authors:** Pricila H. Mullachery, D. Alex Quistberg, Mariana Lazo, Katherine Indvik, Carolina Perez-Ferrer, Nancy López-Olmedo, M. Arantxa Colchero, Usama Bilal

**Affiliations:** 1 Urban Health Collaborative, Drexel Dornsife School of Public Health, 3600 Market St, Philadelphia, PA 19104 USA; 2 Present Address: Department of Health Services Administration and Policy, Temple University College of Public Health, 1301 Cecil B. Moore Ave, Philadelphia, PA 19122 USA; 3 Department of Environmental and Occupational Health, Drexel Dornsife School of Public Health, 3215 Market St, Philadelphia, PA 19104 USA; 4 Department of Community Health and Prevention, Drexel Dornsife School of Public Health, 3215 Market St, Philadelphia, PA 19104 USA; 5 National Council for Science and Technology, 03940 Mexico City, Mexico; 6 Center for Nutrition and Health Research, Instituto Nacional de Salud Pública, 62100 Cuernavaca, Morelos Mexico; 7 Center for Population and Health Research, Instituto Nacional de Salud Pública, 62100 Cuernavaca, Morelos Mexico; 8 Center for Health Systems Research, Instituto Nacional de Salud Pública, 62100 Cuernavaca, Morelos Mexico; 9 Department of Epidemiology and Biostatistics, Drexel Dornsife School of Public Health, 3215 Market St, Philadelphia, PA 19104 USA

**Keywords:** Alcohol control policy, Drink-driving policies, Alcohol-related harm, Road traffic deaths, Mexico, Latin America

## Abstract

**Background:**

Up to a third of global road traffic deaths, and one in five in Mexico, are attributable to alcohol. In 2013, Mexico launched a national sobriety checkpoints program designed to reduce drink-driving in municipalities with high rates of alcohol-related collisions. Our study measured the association between the sobriety checkpoints program and road traffic mortality rates in 106 urban municipalities.

**Methods:**

We leveraged data from the Salud Urbana en America Latina (SALURBAL), which compiles health and environmental data from cities with over 100,000 residents. Death data from 2005 to 2019 (i.e., outcome) were from official vital statistics. Among 106 Mexican municipalities defined as priority areas for intervention, 54 adopted the program (i.e., treatment) in 2013, 16 municipalities did so in 2014, 16 in 2015, 10 in 2016, 7 in 2017, and 2 in 2019. We used a difference-in-difference approach with inverse probability weighting adapted to a context where program adoption is staggered over time.

**Results:**

There was a 12.3% reduction in road traffic fatalities per 10,000 passenger vehicles in the post-treatment period compared to the pre-treatment period (95% Confidence Interval, − 17.8; − 6,5). There was a clear trend of decline in mortality in municipalities that adopted the program (vs. comparison) particularly after year 2 of the program.

**Conclusions:**

In this study of 106 municipalities in Mexico, we found a 12.3% reduction in traffic fatalities associated with the adoption of sobriety checkpoints. There was a clear trend indicating that this association increased over time, which is consistent with sustained changes in drink-driving behavior. These findings provide support and insight for efforts to implement and evaluate the impact of sobriety checkpoint policies across Latin America.

**Supplementary Information:**

The online version contains supplementary material available at 10.1186/s40621-022-00407-4.

## Introduction

Road traffic injuries are a major public health problem, representing the eighth most common cause of death globally, and the leading cause for children and young people (5–29 years old) (World Health O 2020). Driving under the influence of alcohol is an important contributor to traffic-related fatalities, with around 30% of traffic-related mortality attributable to alcohol (World Health O 2019) Setting lower limits for blood alcohol content (BAC) and the use of sobriety checkpoints to enforce BAC limits are key drink-driving policies shown to reduce traffic fatalities (Andreuccetti et al. 2011; Erke et al. 2009; Fell and Scherer 2017)

In Mexico, almost 20% of road traffic deaths can be attributed to alcohol consumption (Santoyo-Castillo et al. 2018). Mexico’s national legislation makes it illegal to drive under the influence of alcohol. The BAC limit is set nationally to 0.05 g/dL (Congreso de la 2022), but until May 2022, the BAC limit was 0.08 g/dL in most states, with some states having more strict limits (Pérez-Núñez et al. 2014). For drivers of public transportation vehicles and heavy goods there is a zero-tolerance alcohol policy. However, enforcement of BAC limits depends on local municipalities, which may have limited resources. To improve the enforcement of BAC limits, local and national level policies have been introduced. In 2003, Mexico City created the program “Conduce Sin Alcohol” (CSA), which introduced sobriety checkpoints to monitor the breath alcohol concentration of drivers (Colchero et al. 2020). The CSA program was associated with a reduction in traffic-related deaths of 0.08 per 1 million people per month after the program, compared to the pre-intervention trend (Colchero et al. 2020). In 2010, the cities of Guadalajara and Zapopan, in the state of Jalisco, introduced the “Ley Salvavidas,” which imposed penalties for drivers with a BAC level of 0.05 g/dL or higher and greater penalties for drivers with a higher BAC. This program was associated with a 5.7% decrease in alcohol-related traffic deaths program (Gómez-García et al. 2014).

Following the success of these interventions by local jurisdictions, Mexico launched a national program, the *Programa Nacional de Alcoholimetría* (PNA), or national breathalyzer program, in partnership with the Pan American Health Organization. (CONAPRA. Programa Nacional de Alcoholimetria 2010). This program was designed to support local jurisdictions with high rates of alcohol-related motor vehicle collisions, i.e., priority municipalities, in implementing random and fixed sobriety checkpoints. Under this program, drivers are randomly stopped and those suspected of having consumed alcohol are asked to take an alcohol breath test. If alcohol is detected, further tests are administered to measure the BAC, and if the blood alcohol level is above the legal limit or a driver refuses to take a test (less common), they face fines and may be detained for up to 24 h.

The existence of sobriety checkpoints that are well publicized can work as a deterrent to drink-driving (Fell et al. 2004, 2008; World Health O 2019). A review of the literature found that sobriety checkpoints are effective in reducing traffic fatalities, but reductions varied from 8 to 71% in different studies (Erke et al. 2009). In addition to deterring people from driving while intoxicated, which may reduce alcohol-related collisions, checkpoint programs may also interact with other road safety policies because awareness of checkpoints may change drivers’ behavior regarding seatbelt use or speeding. This is likely to contribute to safer roads for all drivers and overall reductions in traffic fatalities. Most of the literature on the effectiveness of drink-driving policies comes from high-income countries, while less evidence is available for low- and middle-income countries in general, and Latin American countries in particular (Babor and Caetano 2005). To better understand how sobriety checkpoints may impact traffic-related fatalities, research evaluating the effect of these policies in different contexts is needed.

The road safety policy environment in Mexico is complex; state and local governments have the power to enact road safety policies. An analysis of legislation enacted up until 2016 found that drink-driving, speeding, and seatbelt use legislation are insufficient or inadequate in most states (Pérez-Núñez et al. 2017). The adoption of a well-publicized national sobriety checkpoints program has the potential to improve road safety and reduce the burden of traffic fatalities in Mexico. The objective of this study is to measure the association between sobriety checkpoints and traffic-related mortality in highly populated urban municipalities with high rates of alcohol-related traffic collisions in Mexico. We examine this association immediately after the adoption of the program and over time, for six years, to assess whether the relationship is sustained over time.

## Methods

### Study design and sample

This is a quasi-experimental study measuring the effect of sobriety checkpoints on traffic-related fatalities. We used data from the Salud Urbana en America Latina (SALURBAL), which compiles health and environmental data from all cities with more than 100,000 residents in 11 Latin American countries (Bilal et al. 2021; Diez Roux et al. 2019; Quistberg et al. 2022, 2019). For more details about the SALURBAL project, see https://drexel.edu/lac/salurbal. The present study included data from 106 municipalities defined as priority municipalities by Mexico’s Ministry of Health for implementation of sobriety checkpoints. Priority municipalities were defined as those with higher proportions of alcohol-related motor vehicle collisions, compared with the national average, between 2010 and 2012. (CONAPRA. Acción Estratégica de Alcoholimetría 2013). Originally, 128 municipalities were defined as priority municipalities, but among these, only 106 are included in the SALURBAL data resource for which we had outcome data. Our final sample consisted of 106 municipalities that together represent 46% of Mexico’s population.

### Treatment variable

We used data from the National Council for Injury Prevention (CONAPRA) containing the list of municipalities that adopted the program and the year of adoption. Unfortunately, data on the number of sobriety checkpoints in each municipality were not available, but municipalities are likely to have several sobriety checkpoints, particularly during weekends and holidays when people are more likely to consume alcohol (VDN Noticias 2016; Oaxaca Entrelineas 2014; Hernández 2016). Among the 106 municipalities in the study, 54 adopted the program in 2013, 16 in 2014, 16 in 2015, 10 in 2017, and 2 in 2019. One municipality did not adopt the program within the study period. A small number of municipalities adopted the program and later discontinued it. More on this issue is discussed in the analytic strategy (sensitivity analysis).

### Outcome variable

The outcome of interest was the yearly rate of road traffic mortality from 2005 to 2019, calculated as the number of road traffic deaths per 10,000 registered passenger vehicles. Mortality data were obtained from official vital statistics data, and road traffic deaths were defined as those with codes V01-V89 of the International Classification of Diseases, 10th revision (ICD-10), as the underlying cause of death with a few exceptions (Additional file 1: Table 1). We included all motor vehicle transportation deaths, whether they were classified as traffic-related or not, because the small share (less than 1%) of deaths classified as non-traffic deaths could still capture traffic-related deaths in parking lots, driveways, private roads, or parks. We corrected for underreporting of deaths using an ensemble of death-distribution methods described elsewhere (Bilal et al. 2021). In order to correct for miscoding of road traffic deaths as ill-defined deaths (e.g., X59), we used established methods (Bhalla et al. 2009a, 2009b) from prior literature to redistribute ill-defined codes to specifically defined and partially defined codes (Quistberg et al. 2022). The number of registered passenger vehicles was obtained from the Instituto Nacional de Estadística y Geografía (INEGI), (Vehículos de motor registrados en circulación 2020) which reports these data publicly on an annual basis.

### Covariates

We included pre-treatment covariates that represent broad characterizations of the form of the municipality that may be associated with road traffic deaths (Quistberg et al. 2022): 1) total population in 2012; 2) a measure of urban development fragmentation (i.e., patch density) defined as the number of continuous urban development patches per 100 square kilometers of land in 2012; 3) percent urban “built-up,” defined as the percentage of the land area of the municipality covered by urban patches in 2012; and 4) a socioeconomic score constructed using the proportion of the population aged 25 or older with secondary education and college education completed in 2010. Data were compiled from various sources as part of the SALURBAL project. Details about these metrics are described elsewhere (Quistberg et al. 2019; Ortigoza et al. 2021).

### Analytical strategy

We first described medians and interquartile ranges of road traffic fatalities per 10,000 passenger vehicles during pre- and post-treatment periods (i.e., before and after program adoption) for municipalities grouped by year of adoption, such that for municipalities that adopted the program in 2013 (n = 54), the pre-treatment period was 2005–2012 and the post-treatment period was 2013–2019. Likewise, for municipalities that adopted the program in 2014 (n = 16), the pre-treatment period was 2005–2013 and the post-treatment period was 2014–2019, and so forth. We also graphically examined covariate balance across municipalities by treatment group.

We used a difference-in-difference (DiD) framework with multiple time periods and variations in treatment timing, as proposed by (Callaway and Sant’Anna 2021). The simplest DiD design compares two groups, i.e., treated and comparison groups (first difference), in two periods, before and after the treatment (second difference). The approach developed by (Callaway and Sant’Anna 2021) uses the classic DiD framework to compare multiple groups over multiple time periods and then estimates a global effect by combining weighted averages of group-time treatment effects. This approach is recommended when units receive the treatment at different time points, i.e., staggered treatment adoption, to avoid bias previously reported with traditional DiD approaches such as two-way fixed effect models (Athey and Imbens 2021; Sun and Abraham 2021).

In summary, this method compares the group treated in a particular year (e.g., 2013) with the groups that will receive the treatment in subsequent years (e.g., 2014–2019) at different points in time to estimate group-time specific effects. In this setup, the comparison group for each period changes over time; for example, for the group treated in 2013, the comparison group in 2013 contains all units that were not treated in 2013 (i.e., not-yet-treated in 2013). This logic is repeated for all other groups treated subsequently, with the comparison groups containing all units that were not yet treated. To estimate the group-time average treatment effect on the treated (ATTs), we used linear regression models adjusted for pre-treatment covariates combined with inverse probability weighting (IPW). IPWs are based on propensity scores, which are used to create weights that balance treated and comparison groups with respect to the covariates and reduce the likelihood of confounding bias. This combination of IPW in covariate-adjusted models, known as a doubly robust model, is a powerful tool that allows for two opportunities to correctly specify the model without introducing additional bias (Sant’Anna and Zhao 2020). We used the same set of covariates for adjustment and construction of the IPWs. Finally, the models also accounted for the clustering of municipalities into larger metropolitan regions.

We presented results for the global effect estimate of the treatment, i.e., the global ATT, on the outcome, which is a weighted average of all group-time ATTs. We also presented dynamic treatment effect, which represents the effect of the treatment at various points in time, e.g., one year or three years after the treatment.

We included several sensitivity analyses with the goal of testing for potential biases. To isolate the effect of sobriety checkpoints from prior drink-driving policies (Colchero et al. 2020), we excluded municipalities that are part of Mexico City (sensitivity 1). To test for potential heterogeneity in the program (i.e., multiple versions of the treatment), we excluded 17 municipalities for which the program was discontinued during the study period (sensitivity analysis 2). To test for potential spillover effects, i.e., contamination bias, we used larger geographic units (n = 72) representing groups of municipalities that are part of a contiguous metropolitan area (sensitivity analysis 3). The goal of this approach was to account for potential spillover effects from municipalities that adopted the program to municipalities that did not adopt the program but might have been affected by it due to geographic proximity. We tested two different ways of assigning treatment to the larger metropolitan area: 1) with treatment defined according to the first municipality to adopt the program within the larger metropolitan area and 2) with treatment defined according to the core (central) municipality in the larger metropolitan area. Finally, we conducted a negative control analysis (sensitivity analysis 4) where we repeated the primary analysis using cancer deaths, an outcome for which we do not expect a short-term effect from sobriety checkpoints.

All analyses were conducted using STATA v17 and the STATA package CSDID, (StataCorp 2020) developed in collaboration with the authors of Callaway and Sant’Anna (2020).

## Results

**Fig. 1:**
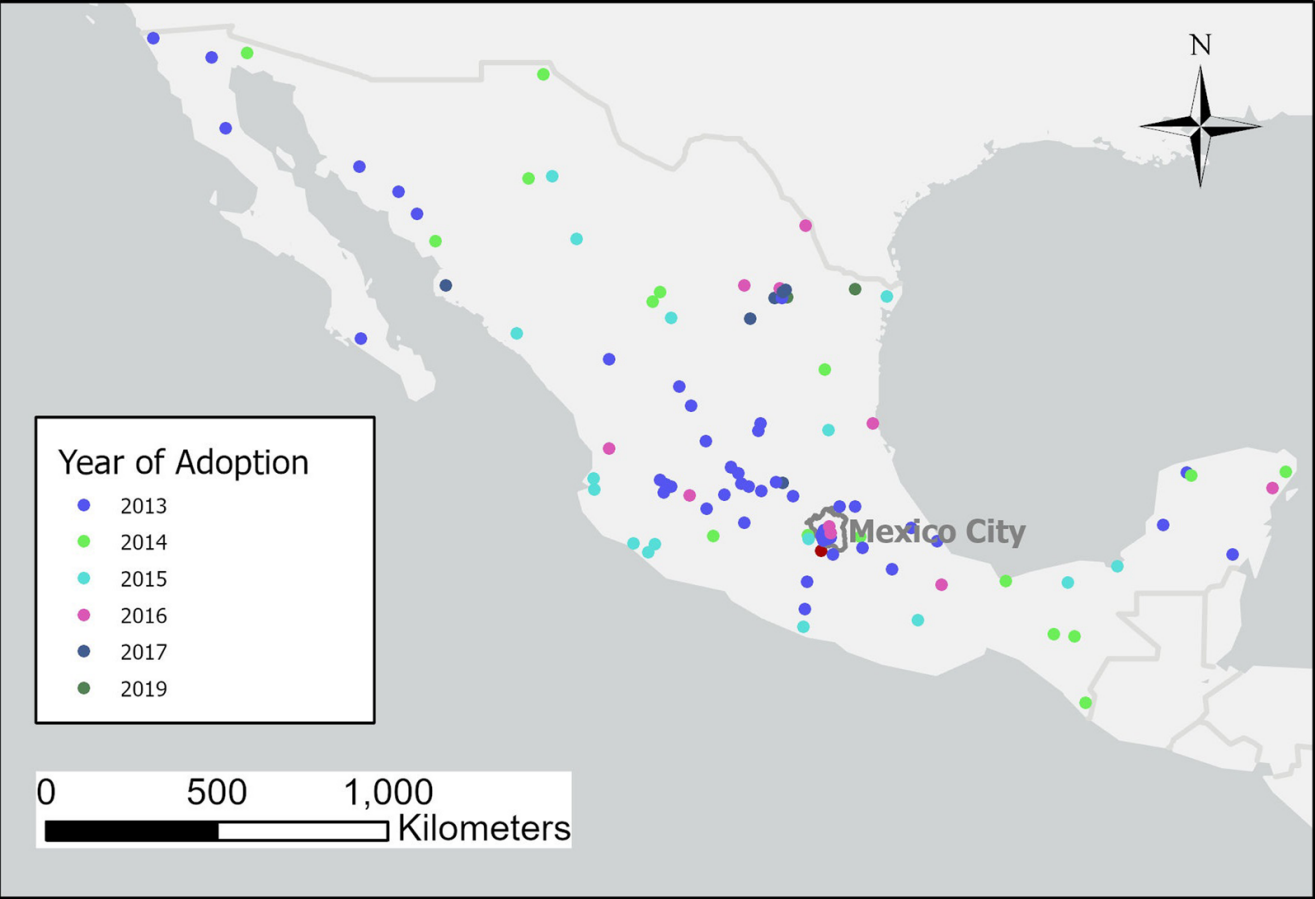
Municipalities included in the study grouped by year of adoption of program

**Fig. 2:**
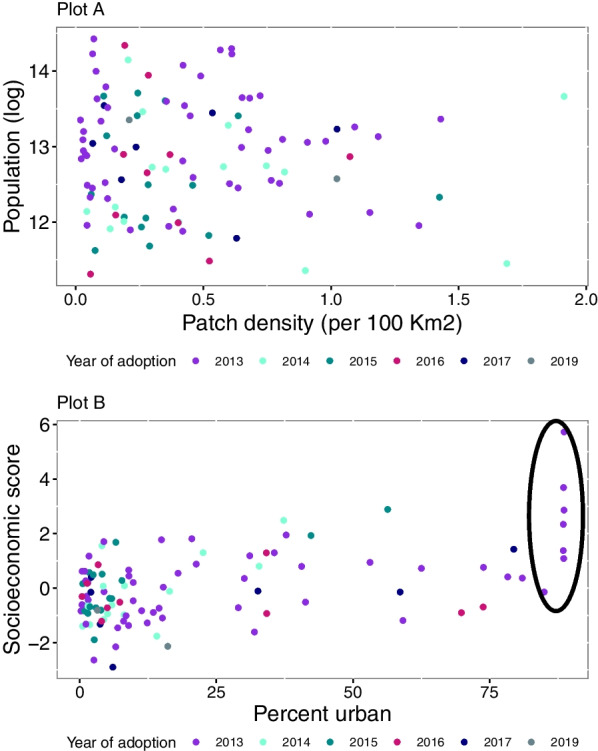
Distribution of municipalities that adopted the program from 2013 to 2019 according to four covariates. Plot A: population size vs. patch density; Plot B: socioeconomic score vs. percent urban. Population is the population in 2012. Patch density is the number of continuous urban development patches per 100 km square of land in 2012. Percent urban is the percentage of the land area of the municipality that is covered by urban patches in 2012. Socioeconomic score is a regression-based score based on the percentage of the population aged 25 or older with secondary education and college completed in 2010. The municipalities outlined in Plot B, adopted the program in 2013, and are unmatched by municipalities in comparison groups. These municipalities were more than 80% urban, and several of them also had high SES scores. These municipalities are part of Mexico City and were excluded in first sensitivity analysis

Among the 106 municipalities in the study, 54 were treated in the first year of the program (2013), and 51 were treated in the subsequent years. Figure 1 shows the location of the municipalities, and Table 1 shows the mortality rates per 10,000 passenger vehicles by group according to year of program adoption. The median mortality rate was slightly higher for the group treated in 2013 than other groups in both pre-treatment (5.76 deaths per 10,000 registered passenger vehicles) and post-treatment (3.61 deaths per 10,000) periods. In all groups, the post-treatment rate was lower than the pre-treatment rate. Figure 2 shows the distribution of covariates (i.e., population, percent urban, patch density, and socioeconomic score) by treatment group. Municipalities were well distributed across groups and covariates, except for a small number of outlier municipalities with high percent urban built-up that adopted the program in 2013. However, these outlier municipalities are part of Mexico City and were excluded in the first sensitivity analysis. The median and interquartile range for the covariates are also shown in Additional file 2: Table 2 of Appendix.

**Table 1:** Descriptive statistics for yearly rates of traffic-related fatalities per 100,000 vehicles registered by treatment group, defined as year of program adoption

Year of adoption	N	Pre-treatment^1^			Post-treatment^1^		
		Median	25th percentile	75th percentile	Median	25th percentile	75th percentile
2013	54	5.76	3.92	11.54	3.61	2.37	7.2
2014	16	5.13	2.65	6.29	3.21	1.85	4.29
2015	16	3.04	2.55	9.32	2.09	1.67	5.68
2016	10	3.38	2.42	6.07	2.42	1.89	3.97
2017	7	4.4	2.57	8.36	2.78	1.71	4.56
2019	2	4.99	1.84	8.14	3.06	1.56	4.57
Did not adopt	1	3.9	3.9	3.9	–	–	–

^1^Pre-treatment and post-treatment periods vary by group. For example, for municipalities that adopted the program in 2013, the pre-treatment period included data from 2005 to 2012 and the post-treatment period included data from 2013 to 2019. For municipalities that adopted the program in 2014, the pre-treatment period included data from 2005 to 2013 and the post-treatment period included the years 2014–2019

Table 2 shows the global effect estimates from the difference-in-difference models comparing treated and not-yet-treated municipalities. In our main analysis, we found an average 12.3% reduction (95% CI, − 17.8; − 6.5), in road traffic fatalities per 10,000 passenger vehicles in the post-treatment period compared to the pre-treatment period. Our sensitivity analyses showed similar results compared to the main analysis. The model excluding municipalities in Mexico City showed a slightly smaller effect of − 8.5% (95% CI, − 13.3; − 3.4); the model excluding cities where the program was discontinued showed an ATT of − 11.9% (95% CI, − 18.1; − 5.2); and the models using larger metropolitan areas (instead of municipalities) showed larger ATTs, but these were accompanied by larger confidence intervals (− 22.9%, 95% CI − 35.1 to − 8.6%, when we assigned treatment based on the first municipality to adopt the program, and − 18.4, 95% CI − 27.2 to − 8.8, when we assigned treatment based on the core municipality). Finally, the model using cancer deaths as a negative control showed no effect of the sobriety checkpoints program on this outcome (ATT = 1.2%,95% CI − 2.9–5.6%).

**Table 2:** Average treatment effect on the treated (ATT) of sobriety checkpoints on rates of traffic fatalities in Mexican municipalities

Model	ATT (95% CI)
Main model (n = 106)	− 12.3% (− 17.8;− 6.5)
Sensitivity 1: excluding Mexico City (n = 92)	− 8.5% (− 13.3;− 3.4)
Sensitivity 2: excluding municipalities where the program was discontinued (n = 89)	− 11.9% (− 18.1;− 5.2)
Sensitivity 3.1: using metropolitan areas and first municipality to adopt (n = 72)	− 22.9% (− 35.1;− 8.6)
Sensitivity 3.2: using metropolitan areas and core municipality (n = 72)	− 18.4% (− 27.2;− 8.8)
Sensitivity 4: negative control (cancer deaths as outcome) (n = 106)	1.2% (− 2.9;5.6)

Global effect estimates were exponentiated and calculated as a percent change, calculated as (exp(B)-1)*100. Models were adjusted for pre-treatment covariates combined with inverse probability weighting (IPW). In models using municipality-level data (i.e., main model and sensitivity models 1, 2, and 4), municipalities were clustered within metropolitan areas using clustered robust standard errors

**Fig. 3:**
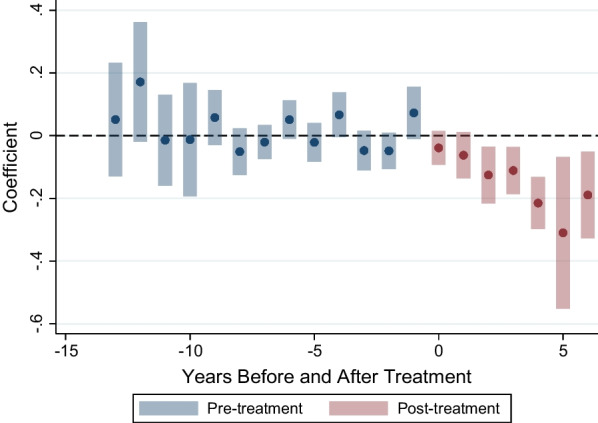
Dynamic treatment effect (ATT, 95% CI) of sobriety checkpoints on traffic mortality rates. The coefficients represent the weighted average of the group-time differences in outcome among treated and not-yet-treated groups in the years pre- and post-adoption. The not-yet-treated group progressively reduces as more municipalities adopt the program over the years

The dynamic treatment effect specification for the main model is represented in Fig. 3. Pre-treatment estimates and confidence intervals close to zero indicate that there was no differential trend between the groups of municipalities before treatment. However, in the post-treatment period, we see a clear trend indicating that the mortality rate was increasingly lower in treated vs. comparison groups, particularly after year 2 of the program.

## Discussion

In this study examining the effect of sobriety checkpoints on road traffic fatalities per 10,000 passenger vehicles in urban Mexican municipalities, we found that the program was associated with a 12.3% overall reduction in road traffic mortality. Overall, this result supports the hypothesis that sobriety checkpoints are associated with reductions in traffic mortality rates. The dynamic analysis showed that while the program did not have an immediate effect on the outcome, there was a clear trend of decline in road traffic mortality in the treated (vs. comparison) group, particularly after year 2 of the program. Various sensitivity analyses returned consistent results, including a negative control using cancer rates as the outcome.

Our results are generally consistent with the literature assessing the effect of sobriety checkpoint programs (Bergen et al. 2014; Siegfried and Parry 2019; Peek-Asa 1999). Studies have shown reductions in fatal and non-fatal vehicle crashes and alcohol-related collisions. Studies conducted in the 80s and 90s found reductions ranging from 16 to 29% when measuring overall fatalities before and after adoption of similar programs (Peek-Asa 1999). More recent evaluations of sobriety checkpoint programs also found reductions on alcohol-involved crashes, with a median 8.9% reduction across 10 studies in the USA (Bergen et al. 2014). This reduction in effect over time is hypothesized to be the result of already large declines in alcohol-related crashes in previous decades in high-income countries and lower enforcement of these policies over time, particularly in the USA (Fell et al. 2014). Our results indicate that Mexico could be at an intermediate point where larger reductions are still expected, although we did not directly measured alcohol-related crashes. In addition, we did not have data to assess the program’s enforcement and implementation (e.g., number of checkpoints) and therefore we could not account for these dimensions in our analysis. Future studies should further examine this issue by measuring variation in intensity (i.e., number and type of sobriety checkpoints, fines) as well as enforcement (i.e., number of stops and hours of operation) across different cities to better understand effect heterogeneity of the program and its impact on driver behavior and mortality.

Our study found that the program was particularly effective after the first two years, even though more than half of municipalities adopted the program in the first year (2013). Most previous studies evaluating similar programs only followed outcomes for 1–3 years, (Bergen et al. 2014; Peek-Asa 1999; Elder et al. 2002) so less is known about the long-term impact of these programs. Our results suggest that the Mexican sobriety checkpoints program led to sustained behavior change among drivers. Our findings provide support for future research examining these longer-term effects and the conditions needed to create and maintain desired changes.

Our study has several advantages compared to previous evaluations of sobriety checkpoints. First, we included a large number of jurisdictions with high rates of road traffic fatalities at baseline that adopted the program over a number of years. We took advantage of this large sample and the staggered adoption of the program across the country to compare jurisdictions that adopted the program with those that would eventually adopt the program. The inclusion of such a comparison group strengthened our study design by reducing the chances of unmeasured confounders, i.e., covariates associated with both the adoption of the program and the outcome. Second, we used a combination of regression adjustment and inverse probability weights, which together improve the plausibility of the conditional parallel trends assumption holding true for municipalities with similar characteristics, e.g., population size and urban development. Third, municipalities were well distributed across the range of values of the covariates, except for a handful of outlier municipalities treated in 2013 that had more than 80% urban built-up. These outlier municipalities could lead to positivity violation, also known as lack of overlap, which means that these observations have no counterfactual in the comparison group (Gelman and Hill 2007). However, these outlier municipalities were excluded in the first sensitivity analysis as they are all part of Mexico City. Lastly, we included a number of sensitivity analyses that showed robustness to various analytic decisions. We found that in the analysis excluding Mexico City, the magnitude of the association was smaller compared to the main analysis. This may indicate that Mexico City had better enforcement or higher density of sobriety checkpoints compared to other municipalities. In the sensitivity analysis using larger urban areas, the effect of the program increased dramatically (from 12 to 22%), although confidence intervals were wide (95% CI: 8–25%), potentially due to a smaller sample size (72 large metropolitan areas vs. 106 municipalities). Finally, our negative control (Lipsitch et al. 2010) analysis lends credibility to the claim that these changes are not due to overall changes in mortality or registration of deaths, as we found no association between the program and cancer mortality.

## Limitations

Despite the use of a quasi-experimental approach and a comprehensive set of sensitivity analyses, we cannot claim that the reduction in traffic mortality we observed in our study is fully explained by the sobriety checkpoints program. Mexico has made great strides on road safety measures, with state and local governments enacting legislation and strengthening enforcement. However, there is no central database of these policies, and we were not able to account for all potential changes to local policy environments during the study period. In this context, our results regarding the sobriety checkpoints program may be overestimated because they could be the result of the combined effect of sobriety checkpoints and other local policies. Nonetheless, our study findings coincide with prior research on sobriety checkpoints in direction and magnitude, which demonstrate large reductions of road traffic deaths and injuries when checkpoints are implemented and enforced. The lack of available data on the intensity or enforcement of the program across municipalities represents an important limitation of our study. The fact that we did not adjust for these features indicates that our global effect is an average across localities with potentially different levels of intensity or enforcement of the program. In this context, our findings are likely an underestimation of the program’s potential effectiveness if implementation had been homogeneous across locations. We also acknowledge that road traffic mortality is an imperfect proxy for alcohol-related traffic deaths. However, studies indicate that sobriety checkpoints may have a broader effect on road traffic deaths overall by leading to better behavior from drivers in general and improving road safety by removing high-risk drivers from the driver pool. Lastly, vital registration data may be incomplete due to insufficient coverage or ill-defined deaths, although we took steps to mitigate both of these issues (Quistberg et al. 2022).

## Conclusions

In this study of 106 municipalities in Mexico, we found a 12.3% reduction in traffic mortality rate associated with the adoption of sobriety checkpoints. There was a clear trend indicating that this association increased over time, particularly after year 2 of the checkpoint program. These findings provide support for efforts to implement sobriety checkpoint programs in Latin America, and insights for assessing similar initiatives implemented to reduce alcohol-related harms.

## Supplementary Information

**Additional file 1.**. ICD-10 codes excluded in the identification of road traffic deaths

**Additional file 2**. Descriptive statistics for covariates by treatment group.

## Data Availability

The datasets analyzed during the current study are available. Mortality data for Mexico were downloaded from publicly available repositories available here: https://www.inegi.org.mx

## Abbreviations

BAC: Blood alcohol content
CSA: Conduce sin alcohol
PNA: Programa Nacional de Alcoholimetría
SALURBAL: Salud Urbana en America Latina
ICD-10: International Classification of Diseases, 10th revision
CONAPRA: National Council for Injury Prevention (English)
INEGI: Instituto Nacional de Estadistica y Geografia
DID: Difference-in-difference
ATT: Average treatment effect on the treated
IPW: Inverse probability weighting
CI: Confidence interval

## References

[CR1] Andreuccetti G, Carvalho HB, Cherpitel CJ (2011). Reducing the legal blood alcohol concentration limit for driving in developing countries: a time for change? Results and implications derived from a time–series analysis (2001–10) conducted in Brazil. Addiction.

[CR2] Athey S, Imbens GW (2021). Design-based analysis in difference-in-differences settings with staggered adoption. J Econom.

[CR3] Babor TF, Caetano R (2005). Evidence-based alcohol policy in the Americas: strengths, weaknesses, and future challenges. Rev Panam Salud Publ.

[CR4] Bergen G, Pitan A, Qu S (2014). Publicized sobriety checkpoint programs: a community guide systematic review. Am J Prev Med.

[CR5] Bhalla K, Shahraz S, Bartels D, Abraham J (2009). Methods for developing country level estimates of the incidence of deaths and non-fatal injuries from road traffic crashes. Int J Inj Contr Saf Promot.

[CR6] Bhalla K, Naghavi M, Shahraz S, Bartels D, Murray CJL (2009). Building national estimates of the burden of road traffic injuries in developing countries from all available data sources: Iran. Inj Prev.

[CR7] Bilal U, Hessel P, Perez-Ferrer C (2021). Life expectancy and mortality in 363 cities of Latin America. Nat Med.

[CR8] Callaway B, Sant’Anna PHC (2021). Difference-in-differences with multiple time periods. J Econom.

[CR9] Colchero MA, Guerrero-López CM, Quiroz-Reyes JA, Bautista-Arredondo S (2020). Did, “Conduce Sin Alcohol” a program that monitors breath alcohol concentration limits for driving in Mexico City Have an effect on traffic-related deaths?. Prev Sci.

[CR10] CONAPRA. Acción Estratégica de Alcoholimetría. 2013. http://conapra.salud.gob.mx/Interior/Linea_Estrategica_Alcoholimetria.html.

[CR11] CONAPRA. Programa Nacional de Alcoholimetria. 2010. p. http://conapra.salud.gob.mx/Interior/Documentos/Manuales/Programa_Nacional_Alcoholimetria.pdf.

[CR12] Congreso de la Union. Ley General de Movilidad y Seguridad Vial. https://www.diputados.gob.mx/LeyesBiblio/pdf/LGMSV.pdf; 2022.

[CR13] Diez Roux AV, Slesinski SC, Alazraqui M (2019). A novel international partnership for actionable evidence on urban health in Latin America: LAC-urban health and SALURBAL. Glob Chall.

[CR14] Elder RW, Shults RA, Sleet DA, Nichols JL, Zaza S, Thompson RS (2002). Effectiveness of sobriety checkpoints for reducing alcohol-involved crashes. Traffic Inj Prev.

[CR15] Erke A, Goldenbeld C, Vaa T (2009). The effects of drink-driving checkpoints on crashes—a meta-analysis. Accid Anal Prev.

[CR16] Fell JC, Scherer M (2017). Estimation of the potential effectiveness of lowering the blood alcohol concentration (BAC) limit for driving from 008 to 005 grams per deciliter in the United States. Alcohol Clin Exp Res.

[CR17] Fell JC, Lacey JH, Voas RB (2004). Sobriety checkpoints: evidence of effectiveness is strong, but use is limited. Traffic Inj Prev.

[CR18] Fell JC, Waehrer G, Voas RB, Auld-Owens A, Carr K, Pell K (2014). Effects of enforcement intensity on alcohol impaired driving crashes. Accid Anal Prev.

[CR19] Fell JC, Tippetts AS, Levy M. Evaluation of seven publicized enforcement demonstration programs to reduce impaired driving: Georgia, Louisiana, Pennsylvania, Tennessee, Texas, Indiana, and Michigan. 2008: Association for the Advancement of Automotive Medicine. p. 23. PMC3256786 19026220

[CR20] Gelman A, Hill J. Causal inference using more advanced models. In Data analysis using regression and multilevel/hierarchical models (1st ed). New York: Cambridge University Press; 2007. p. 215–26.

[CR21] Gómez-García L, Pérez-Núñez R, Hidalgo-Solórzano E (2014). Impacto de la reforma en la legislación sobre consumo de alcohol y conducción en Guadalajara y Zapopan, Jalisco, México: una mirada en el corto plazo. Cad Saude Publ.

[CR22] Hernández E. Operativo Alcoholímetro las 24 horas del día: SSP CDMX. La Prensa. 2016. https://wwwla-prensacommx/metropoli/operativo-alcoholimetro-las-24-horas-del-dia-ssp-cdmx-3502517html.

[CR37] INEGI. Registered motor vehicles in circulation. 2020. Available at: http://en.www.inegi.org.mx/programas/vehiculosmotor.

[CR23] Lipsitch M, Tchetgen ET, Cohen T (2010). Negative controls: a tool for detecting confounding and bias in observational studies. Epidemiology.

[CR24] Oaxaca Entrelineas. 2014. https://oaxacaentrelineas.com/activan-operativo-“alcoholimetro”-este-31-de-diciembre-y-1-de-enero-enoaxaca/#.Y3UYfC1h10t. Online newspaper: Oaxaca Entrelineas.

[CR25] VDN Noticias. 2016. https://vadenuez.info/wp/colima-aplicara-alcoholimetro-de-forma-permanente/. Online newspaper: VDN Noticias.

[CR26] Ortigoza AF, Granados JAT, Miranda JJ (2021). Characterising variability and predictors of infant mortality in urban settings: findings from 286 Latin American cities. J Epidemiol Commun Health.

[CR27] Peek-Asa C (1999). The effect of random alcohol screening in reducing motor vehicle crash injuries. Am J Prev Med.

[CR28] Pérez-Núñez R, Híjar M, Celis A, Hidalgo-Solorzano E (2014). Road traffic injuries in Mexico: evidences to strengthen the Mexican road safety strategy. Cad Saude Publ.

[CR29] Pérez-Núñez R, Ruelas-Valdés D, Hijar M (2017). Legislación sobre seguridad vial en México: un análisis subnacional. Rev Panam Salud Publ.

[CR30] Quistberg DA, Diez Roux AV, Bilal U (2019). Building a data platform for cross-country urban health studies: the SALURBAL study. J Urban Health.

[CR31] Quistberg DA, Hessel P, Rodriguez DA (2022). Urban landscape and street-design factors associated with road-traffic mortality in Latin America between 2010 and 2016 (SALURBAL): an ecological study. Lancet Planet Health.

[CR32] Sant’Anna PHC, Zhao J (2020). Doubly robust difference-in-differences estimators. J Econom.

[CR33] Santoyo-Castillo D, Pérez-Núñez R, Borges G, Híjar M (2018). Estimating the drink driving attributable fraction of road traffic deaths in Mexico. Addiction.

[CR34] Siegfried N, Parry C (2019). Do alcohol control policies work? An umbrella review and quality assessment of systematic reviews of alcohol control interventions (2006–2017). PLoS ONE.

[CR35] StataCorp (2020). Stata Statistical Software: Release 16.

[CR36] Sun L, Abraham S (2021). Estimating dynamic treatment effects in event studies with heterogeneous treatment effects. J Econom.

[CR38] World Health O. Global status report on alcohol and health 2018b: World Health Organization; 2019.

[CR39] World Health O. The SAFER technical package: five areas of intervention at national and subnational levels. 2019.

[CR40] World Health O. Global status report on road safety 2018a. december 2018a. 2020.

