# Supplementary material for: Evaluation of the national sobriety checkpoints program in Mexico: a difference-in-difference approach with variation in timing of program adoption
Source: Inj Epidemiol. 2022 Nov 21;9:32. doi: 10.1186/s40621-022-00407-4 (PMC9680121; doi:10.1186/s40621-022-00407-4)
Supplement: Supplementary file 1 — Additional file 1.. ICD-10 codes excluded in the identification of road traffic deaths [file 40621_2022_407_MOESM1_ESM.docx]

**Supplemental Table 1: ICD-10 codes excluded in the identification of road traffic deaths**

| V81.2 – Occupant of railway train or railway vehicle injured in collision with or hit by rolling stock  V81.3 – Occupant of railway train or railway vehicle injured in collision with other object  V81.4 – Person injured while boarding or alighting from railway train or railway vehicle  V81.5 – Occupant of railway train or railway vehicle injured by fall in railway train or railway  vehicle  V81.6 – Occupant of railway train or railway vehicle injured by fall from railway train or railway vehicle  V81.7 – Occupant of railway train or railway vehicle injured in derailment without antecedent collision  V81.8 – Occupant of railway train or railway vehicle injured in other specified railway accidents V81.9 – Occupant of railway train or railway vehicle injured in unspecified railway accident. |
| --- |
