# Supplementary material for: Evaluation of the national sobriety checkpoints program in Mexico: a difference-in-difference approach with variation in timing of program adoption
Source: Inj Epidemiol. 2022 Nov 21;9:32. doi: 10.1186/s40621-022-00407-4 (PMC9680121; doi:10.1186/s40621-022-00407-4)
Supplement: Supplementary file 2 — Additional file 2. Descriptive statistics for covariates by treatment group. [file 40621_2022_407_MOESM2_ESM.docx]

**Supplemental Table 2: Descriptive statistics for covariates by treatment group.**

| Treatment Year | Population (in 100,000) | | Percent urban | | Patch density (per 100 km^2^) | | Socioeconomic Index | |
| --- | --- | --- | --- | --- | --- | --- | --- | --- |
|  | Median | 25th - 75th | Median | 25th - 75th | Median | 25th - 75th | Median | 25th - 75th |
| Treated in 2013 | 4.74 | 2.71 - 8.04 | 14.5 | 4.44-40.58 | 0.42 | 0.08-0.68 | 0.24 | -0.83 - 0.94 |
| Treated in 2014 | 3.22 | 1.63 - 4.64 | 5.48 | 2.93-15.25 | 0.38 | 0.2-0.78 | -0.59 | -1.07 - 0.44 |
| Treated in 2015 | 2.35 | 1.52 - 6.66 | 2.96 | 1.75-5.84 | 0.26 | 0.12-0.35 | -0.11 | -0.77 - 0.54 |
| Treated in 2016 | 3.87 | 1.61 - 3.99 | 7.32 | 3.36-34.2 | 0.28 | 0.19-0.4 | -0.61 | -0.9 - 0.17 |
| Treated in 2017 | 4.61 | 2.85 - 6.91 | 6.05 | 2.08-58.61 | 0.24 | 0.11-0.63 | -0.15 | -1.33 - 0.39 |
| Treated in 2019 | 4.59 | 2.89 - 6.3 | 9.66 | 3.22-16.1 | 0.62 | 0.21-1.02 | -1.47 | -2.14 - -0.81 |
| Total | 3.92 | 2.22 - 6.66 | 8.17 | 2.59-32.6 | 0.36 | 0.13-0.64 | -0.14 | -0.84 - 0.8 |
